# Neonatal Disseminated Herpes Simplex Virus Infection Triggering Extreme Hyperferritinemia Concerning for Hemophagocytic Lymphohistiocytosis

**DOI:** 10.1177/2324709619862840

**Published:** 2019-07-18

**Authors:** Grace Averitt, Mohamad M. Al-Rahawan, Fatma Levent

**Affiliations:** 1 Texas Tech Health Sciences Center, Lubbock, TX, USA

To the Editor,

Neonatal herpes simplex virus (HSV) is often a devastating disease especially if acquired after a primary maternal infection. Up to 80% of mothers are unaware of their positive herpetic status. Disseminated disease carries the highest mortality with up to 77% death rate after diagnosis. Patients may present with minimal symptoms, such as only fever as in our case, or with central nervous system manifestations and sepsis.^1^ Rarely, HSV infections may trigger underlying inherited immune dysfunction with devastating effect.

Hemophagocytic lymphohistiocytosis (HLH) is a life-threatening syndrome characterized by a reactive process resulting from prolonged and excessive activation of immune cells. The predominant clinical findings include fevers, cytopenias, hepatitis, and splenomegaly.^2^ Multi-organ failure and death are likely if HLH is unrecognized or insufficiently treated.^3^ We present a neonate diagnosed with disseminated neonatal HSV-2 infection and extreme hyperferritinemia who was treated for HLH, but died of multi-organ failure.

**Figure 1.**
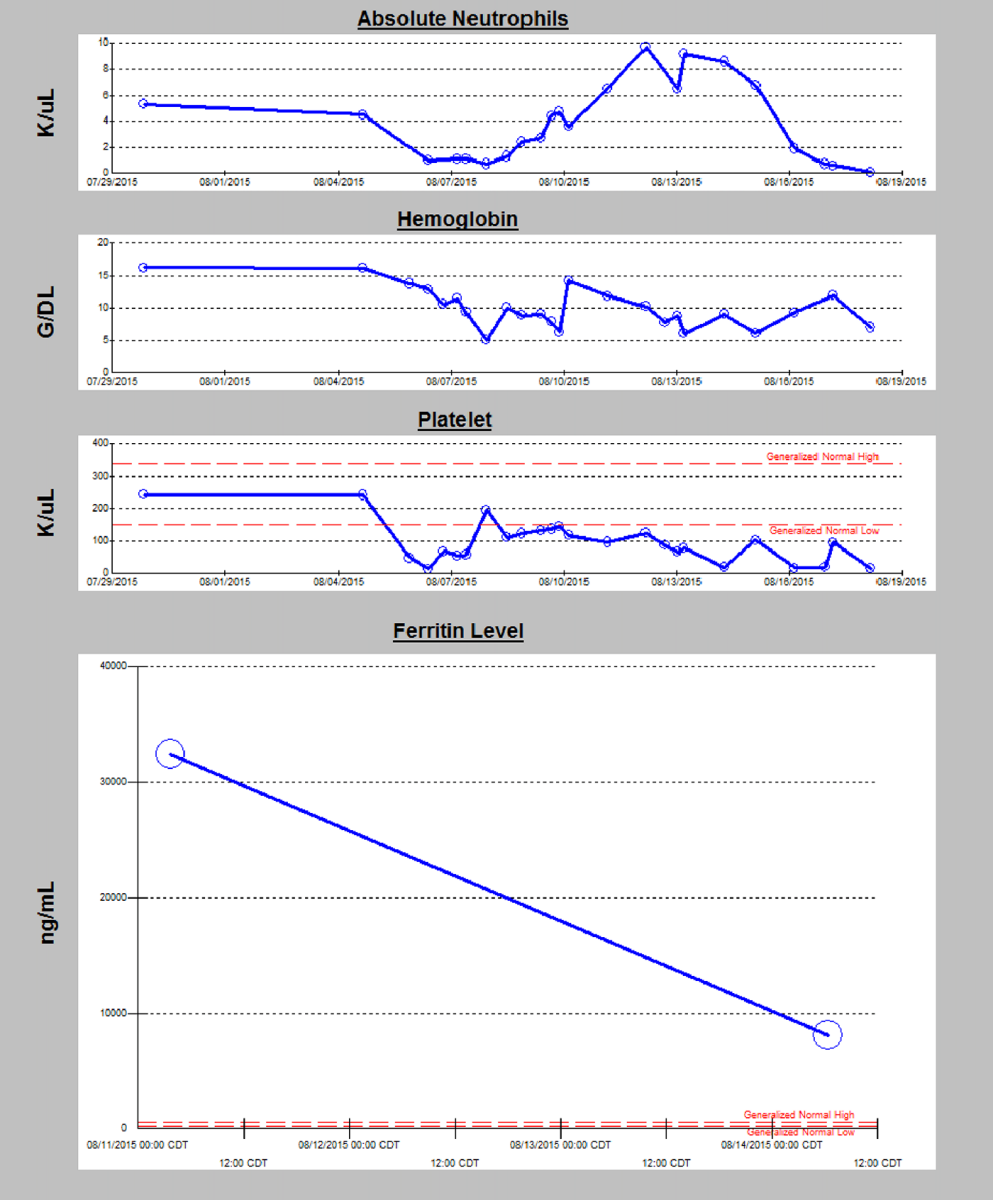
The trends of absolute neutrophils, hemoglobin, platelets, and ferritin during hospitalization.

Our patient was a term male born via Caesarian section to a mother with endometritis. He was admitted with a fever of 101.7°F on the sixth day of life. Ampicillin and cefotaxime were started empirically. He quickly became lethargic with elevated liver function tests, at which time high-dose acyclovir was added. Initial blood counts were normal but severe pancytopenia evolved (Figure 1). He also developed feeding intolerance, hypothermia, and bradycardia with renal, hepatic, and respiratory failure. Blood HSV-2 polymerase chain reaction showed >2 000 000 copies/mL, but cerebrospinal fluid HSV polymerase chain reaction was negative. Disseminated intravascular coagulation and hepatomegaly without splenomegaly were noted. He initially fulfilled only 4 criteria of HLH diagnosis including ferritin >100 000 µg/L, fever, cytopenias, and hypofibrinogenemia.^4^ Bone marrow did not reveal hemophagocytosis or leukemia. Soluble IL-2 and NK cell activity resulted within normal limits.

Dexamethasone was started and etoposide was added later to empirically treat suspected HLH. Multi-organ failure worsened despite maximal support and he died 15 days after admission. Molecular studies for familial HLH revealed variants of unknown significance in 2 of the genes with reference sequences LYST (NM_000081.2) and ITK (NM_005546.3). Family declined an autopsy.

Disseminated neonatal HSV is usually fatal despite appropriate treatment.^4^ Some cases trigger exaggerated inflammatory response that cannot be distinguished from HLH even if elevated ferritin level greater than 10 000 µg/L is more than 90% sensitive and 96% specific for HLH.^5^ Untreated HLH is equally dangerous and is hard to diagnose.^2,6^ Also, it is not easy to justify the use of anti-cytokine therapy in presence of disseminated neonatal HSV. Maeba et al successfully treated neonatal HSV and HLH using a combination of antiviral therapy and methylprednisolone.^7^ Otsubo et al also treated a neonate with HSV complicated by HLH with dexamethasone palmitate successfully.^8^ In contrast, we utilized a combination of antiviral and conventional HLH therapy.

Vladescu et al highlight the conundrum of diagnosis and treatment of HLH in neonates with confirmed disseminated HSV infection and massive hyperferritinemia.^9^ These neonates typically experienced a complex life-threatening course with multi-organ failure. The resulting paradox is extremely challenging and decision of starting HLH treatment should be considered on a case by case basis to avoid unnecessary immunosuppression.
